# Characterization of a Myeloid Activation Signature That Correlates with Survival in Melanoma Patients

**DOI:** 10.3390/cancers12061431

**Published:** 2020-05-31

**Authors:** Mirela Kremenovic, Nives Rombini, Alfred A. Chan, Thomas Gruber, Lukas Bäriswyl, Delphine J. Lee, Mirjam Schenk

**Affiliations:** 1 Institute of Pathology, Experimental Pathology, University of Bern, 3008 Bern, Switzerland; mirela.kremenovic@pathology.unibe.ch (M.K.); nives.rombini@gmail.com (N.R.); thomas.gruber@pathology.unibe.ch (T.G.); lukas.baeriswyl@pathology.unibe.ch (L.B.)); 2 Division of Dermatology, Department of Medicine, The Lundquist Institute at Harbor-UCLA Medical Center, Torrance, CA 90502, USA; alfred.chan@lundquist.org (A.A.C.); delphine.lee@lundquist.org (D.J.L.)

**Keywords:** myeloid cells, melanoma, prognostic gene signature, tumor immunity, innate immunity

## Abstract

Understanding the cellular interactions within the tumor microenvironment (TME) of melanoma paved the way for novel therapeutic modalities, such as T cell-targeted immune checkpoint inhibitors (ICI). However, only a limited fraction of patients benefits from such therapeutic modalities, highlighting the need for novel predictive and prognostic biomarkers. As myeloid cells orchestrate the tumor-specific immune response and influence the efficacy of ICI, assessing their activation state within the TME is of clinical relevance. Here, we characterized a myeloid activation (MA) signature, comprising the three genes *Cxcl11*, *Gbp1*, and *Ido1*, from gene expression data of human myeloid cells stimulated with poly(I:C) or cGAMP. This MA signature positively correlated to overall survival in melanoma. In addition, increased expression of the MA signature was observed in melanoma patients responding to ICI (anti-PD-1), as compared to non-responders. Furthermore, the MA signature was validated in the murine B16F10 melanoma model where it was induced and associated with decreased tumor growth upon intratumoral administration of poly(I:C) and cGAMP. Finally, we were able to visualize co-expression of the MA signature genes in myeloid cells of human melanoma tissues using RNAscope in situ hybridization. In conclusion, the MA signature indicates the activation state of myeloid cells and represents a prognostic biomarker for the overall survival in melanoma patients.

## 1. Introduction

Recent clinical success using immune checkpoint inhibitors (ICI) has demonstrated the therapeutic potential of harnessing T cells to treat various cancer types, including cutaneous melanoma [1,2]. However, only a proportion of patients exhibit durable responses to ICI and certain cancer types remain largely refractory to this line of treatment [1,3].

While it is well known that cytotoxic T cells are a primary effector population in tumor-specific immune responses, the roles of myeloid cells in the tumor microenvironment (TME) are less clear and remain a major focus of investigation [4,5,6]. Tumor-associated myeloid cells represent a vast majority of leukocytes in most tumors and regulate tumor-specific immune responses as well as responses to cancer therapies [5]. Moreover, tumor-associated myeloid cells display significant phenotypic and functional heterogeneity and can either promote or suppress tumor immunity [5,6]. In particular, dendritic cells (DC) and tumor-associated macrophages (TAM) are considered to be key regulators of tumor-specific immune responses due to their function in the priming and recruitment of tumor-infiltrating lymphocytes (TIL) and their ability to modulate tumor stromal and cancer cells [7,8]. As such, a number of therapeutic options such as DC-vaccines and CAR-T cells have been focused on reprogramming intratumoral DC and TAM towards an immunogenic phenotype [7,9,10].

The fact that certain types of human cancer show evidence for spontaneous tumor immunity, prompted investigations to identify innate immune pathways that regulate these responses [11]. Preclinical studies revealed a major role of the stimulator of interferon genes (STING) pathway, a cytosolic DNA sensor, as a crucial event required for optimal type I interferon production, dendritic cell activation, and priming of CD8^+^ T cells against tumor-associated antigens [11,12]. Notably, there is a substantial interest in the investigation of molecules which mimic microbial infection or cellular damage by binding to pattern recognition receptors (PRR) expressed by innate immune cells [13,14]. While a number of PRR agonists are currently undergoing pre-clinical investigations in murine models and some have entered clinical trials, cytosolic PRR for nucleic acids are considered highly attractive targets for the activation of innate immune cells [13,14]. Of particular interest to modulate tumor-specific immune responses are the two PRR, Toll-like receptor 3 (TLR3), which recognizes double stranded RNA and the cytosolic DNA sensor STING [12,14]. TLR3 and STING agonists mimic viral infections, thereby resulting in similar immunological responses such as the production of Type I interferon, the skewing of TAM to an immunogenic phenotype, and promoting maturation and cross-presentation function of DC [4,13]. A number of TLR3 and STING agonists in combination with ICI or other treatments have entered clinical trials to promote an immunogenic TME [14].

In order to select the optimal myeloid cell targeted agents, it is essential to define biomarkers or signatures of activated myeloid cells that are associated with therapeutic response, improved survival, or other clinical parameters. These biomarkers or signatures can then be used in clinic to determine the activation state of myeloid cells and to tailor the treatment regimen. In the context of both DC and TAM, phenotyping requires the assessment of numerous surface and intracellular markers (e.g., CD80, CD86, HLA-DR, CD206, CD163, iNOS, Arg1), to distinguish between pro-inflammatory, immunogenic, and non-inflammatory, immature or immune-suppressive subsets [10,15]. In this study, we describe a 3-gene myeloid activation (MA) signature derived from DC and monocytes treated with the TLR3 agonist poly(I:C) and the bacterial cyclic dinucleotide 3′3′-cGAMP, a STING agonist. The MA signature holds potential for the use in both clinical and pre-clinical applications to determine the presence of activated myeloid cells, such as immunogenic M1 macrophages and to predict the overall survival of melanoma patients.

## 2. Results

### 2.1. Identification of a Myeloid Cell Activation Signature

**Figure 1:**
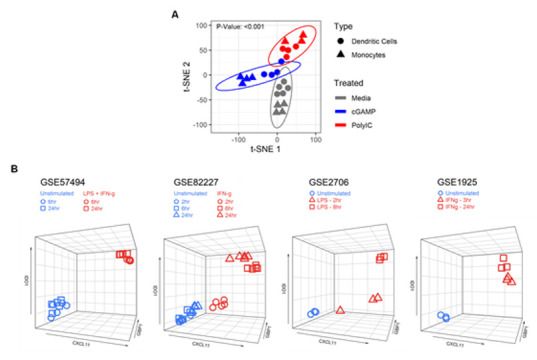
Identification of a three gene signature characteristic to activated myeloid cells. (**A**) Dendritic cells (DC) and CD14^+^ monocytes were isolated from healthy donors and stimulated with poly(I:C) (10 µg/mL) or cGAMP (10 μg/mL) for 6 h in vitro. Gene expression counts were quantified using the NanoString nCounter Immunology Panel. Unsupervised t-Distributed Stochastic Neighbor Embedding (t-SNE) analysis showed significant clustering between unstimulated and stimulated cells for both monocytes and DC. (**B**) IDO1, CXCL11, and GBP1 expression values (RNA-seq, microarray) of unstimulated and stimulated myeloid cells using public Gene Expression Omnibus (GEO) datasets: GSE57494 (LPS + IFNγ), GSE82227 (IFNγ), GSE2706 (LPS), and GSE1925 (IFNγ) plotted as 3D scatter plots using the R package plotly and show clustering between unstimulated and stimulated cells.

To define a human myeloid activation (MA) gene signature, peripheral blood-derived dendritic cells (DC) and CD14^+^ monocytes were isolated from healthy individuals and stimulated with poly(I:C) or cGAMP in vitro. Gene expression profiling was performed using the NanoString nCounter Immunology Panel containing 594 immune-related genes. Unsupervised t-Distributed Stochastic Neighbor Embedding (t-SNE) plot showed significant clustering between unstimulated and stimulated cells for both monocytes and DC (*p*-value: <0.001, Figure 1A).

Further analysis revealed ten differentially expressed genes (DEG) that were shared between myeloid cells stimulated with either poly(I:C) or cGAMP (Table 1).

In order to validate whether these shared genes denote a general state of myeloid cell activation, we analyzed two publicly available microarrays (GSE2706, GSE1925) and two RNA-sequencing (RNA-seq) datasets (GSE57494, GSE82227) that involved human myeloid-derived cells activated with classical innate immune cell stimuli such as LPS and IFNγ. We identified three genes, *Cxcl11*, *Gbp1*, and *Ido1*, that were significantly upregulated in all datasets (Table 2).

Furthermore, 3D scatterplots with *Cxc11*, *Gbp1*, and *Ido1* expression data showed clustering between unstimulated and stimulated cells (Figure 1B), indicating the utility of these three genes as a signature to assess myeloid cell activation.

### 2.2. The MA Gene Signature is Primarily Expressed in Activated Cells of the Myeloid Lineage

**Figure 2:**
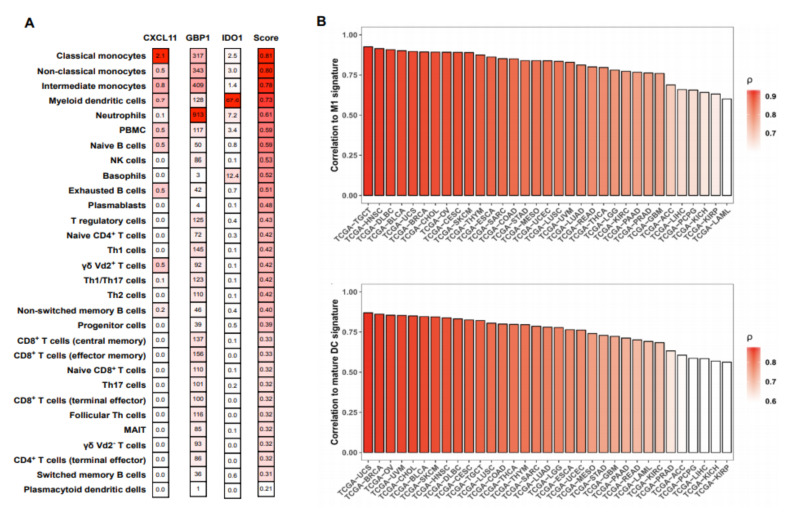
Expression of the myeloid activation (MA) signature genes positively correlated with M1 macrophage and ‘mature DC’ signatures in various cancer types. (**A**) RNA-seq data from peripheral blood mononuclear cells (PBMC) were obtained from GEO (GSE107011) and analyzed for the expression of the signature genes for every cell type available. Transcripts per million (TPM) values were scored with a non-parametric, rank-based method using the R package singscore based on the co-expression of *Cxcl11*, *Gbp1*, and *Ido1.* (**B**) Bulk tumor RNA-seq gene expression data was obtained from The Cancer Genome Atlas (TCGA ) cohorts using GDCRNATools in R. The MA signature score was compared to previously described M1 macrophage (upper panel) and mature DC (lower panel) signatures by Pearson correlation using R. X-axis represents TCGA study abbreviations.

To investigate whether the gene signature (*Cxcl11*, *Gbp1*, and *Ido1*) is specific to activated myeloid cells, we tested whether these genes were upregulated in activated non-myeloid cells (NK cells, B cells, CD8, and CD4 T cells) (GSE63038, GSE85543, GSE79626, GSE60235, respectively). None of the three signature genes was upregulated in activated non-myeloid immune cells, suggesting a selective induction in activated myeloid cells (Supplementary Table S1). Considering the complexity of the immune cell composition in blood and tissue, which can impede the detection of gene signatures, we investigated the expression of our MA signature in RNA-seq data from peripheral blood mononuclear cells (PBMC), comprising 29 distinct immune cells [16]. We found that the baseline expression of *Cxcl11*, *Gbp1*, and *Ido1* in lymphoid cells was lower than in steady-state myeloid cells. Furthermore, the expression of the signature genes was highest in myeloid cells as indicated by a non-parametric, rank-based score (Figure 2A).

Using bulk tumor RNA-seq data from The Cancer Genome Atlas Project (TCGA), we correlated our MA signature to previously described M1 macrophage and mature DC signatures in various cancer types (Supplementary Table S2) [17,18]. The MA signature showed a strong correlation to both the M1 macrophages, as well as to mature DC in all cancer types of the TCGA cohort (Figure 2B).

### 2.3. The MA Gene Signature Correlated with Improved Overall Survival in Melanoma Patients

**Figure 3:**
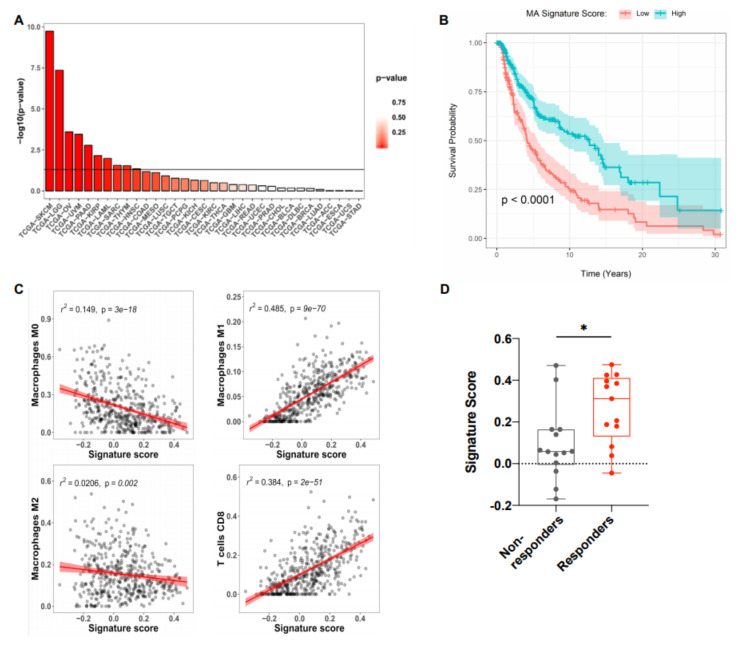
MA signature positively correlates with increased overall survival and the presence of M1 macrophages and CD8^+^ T cells in melanoma. (**A**) RNA-seq data from various tumor types were obtained from TCGA and the MA signature score was assessed with a non-parametric, rank-based method in R using the singscore package. The horizontal line indicates a *p* value of 0.05. (**B**) Kaplan–Meier survival curves of patients with high and low MA signature expression plotted as -log10 *p* values of log-rank tests of survival data for skin cutaneous melanoma (SKCM) with high vs. low MA signature gene expression (split by median) performed in R using the survminer package (*n* = 118). (**C**) Pearson correlation of the MA signature score and the presence of immune cell subsets in SKCM patient data obtained from TCGA. The estimated abundance of various immune cells was determined by Cell-type Identification By Estimating Relative Subsets Of RNA Transcripts (CIBERSORT). Each dot represents an individual patient. (**D**) Signature score in melanoma patients receiving anti-PD-1 (Nivolumab, Pembrolizumab) treatment. Patients were stratified into responders (complete response and partial response, *n* = 13) and non-responders (progressive disease, *n* = 14). Each dot represents an individual patient and only patients sampled pre- and post-treatment were included in the analysis. Dataset was obtained from GSE91061. Box plot defines the maximum, third quartile, first quartile, and minimum values. *p*-values were determined by two-sided Welch’s t-test (* *p* < 0.0332; ** *p* < 0.0021; *** *p* < 0.0002; **** *p* < 0.0001).

Given the important function of M1 macrophages and mature DC within the tumor microenvironment (TME), we investigated the direct association of the MA signature with overall survival (OS) in all cancer types available in TCGA [19]. Survival analysis using log-rank test showed strong association between MA signature and overall survival (OS) in various cancer types (Figure 3A).

In particular, in the skin cutaneous melanoma samples (SKCM), the Kaplan–Meier curve shows that those with higher MA signature scores have consistently higher OS throughout their follow-up (Figure 3B). To further analyze the correlation between the MA signature gene expression and immune cell populations within SKCM, CIBERSORT (Cell-Type Identification By Estimating Relative Subsets of RNA Transcripts) was used to estimate the relative proportions of various immune cell types from bulk RNA-seq data [20]. Tumors with high MA signature expression displayed increased proportions of M1 macrophages, CD8^+^ T cells, and activated CD4^+^ T cells, but reduced M2 and M0 (unpolarized) macrophages (Figure 3C). No additional associations were observed between the MA signature and any of the other immune cell populations investigated (Figure S1). We further examined gene expression data from biopsies of advanced melanoma patients receiving PD-1 blockade therapy. On-treatment biopsies taken from patients with a beneficial response to PD-1 blockade showed increased expression of the MA signature compared to patients with no response to therapy (Figure 3D). Gene expression data from melanoma patients before ICI therapy show a trend of increased signature expression in ICI responders as compared to non-responders (Figure S2). Therefore, the predictive value of this signature for the response to ICI therapy requires further investigation.

### 2.4. Validation of the MA Signature in Murine Myeloid Cells and In Vivo Murine Melanoma

**Figure 4:**
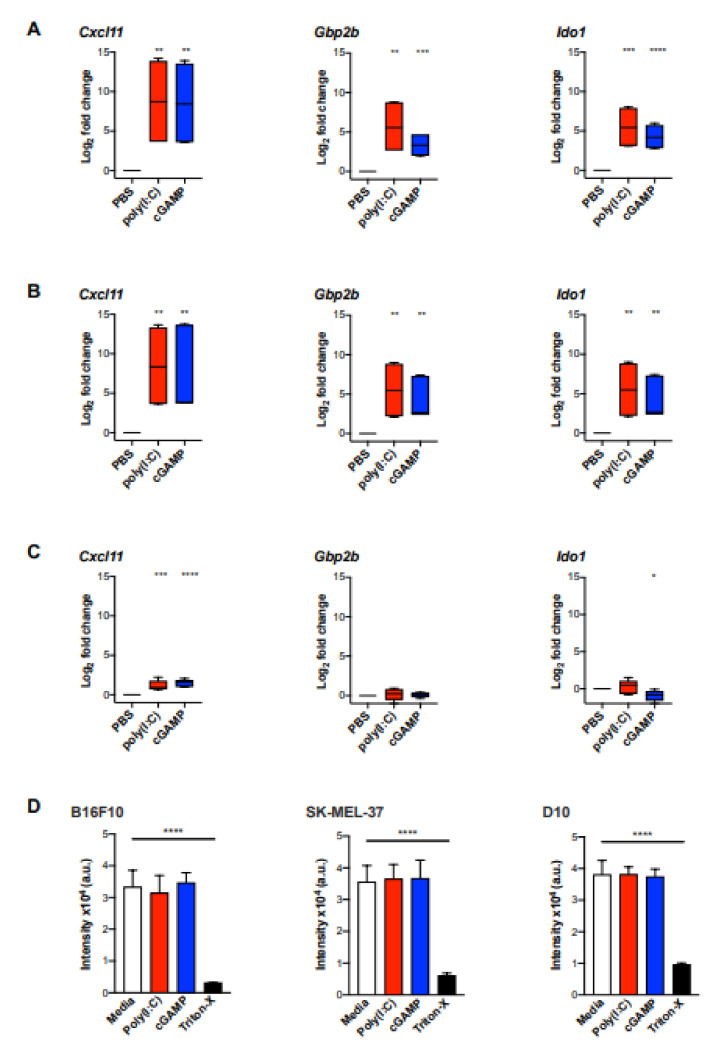
The MA signature expression was significantly induced in bone marrow-derived macrophages (BMDM) but not in melanoma cells upon stimulation with poly(I:C) or cGAMP in vitro. BMDM from three biological replicates were stimulated with poly(I:C) (10 μg/mL) or cGAMP (10 μg/mL) for 6 h (**A**) and 24 h (**B**), following gene expression analysis for *Cxcl11*, *Gbp1*, and *Ido1* by qPCR (*n* = 6). (**C**) B16F10 melanoma cells were cultured with poly(I:C) (10 µg/mL) or cGAMP (10 μg/mL) for 24 h following gene expression analysis by qPCR (*n* = 6). Data are represented as mean ± standard error of log_2_ transformed values. Data were normalized to the housekeeping gene *Rplp0* [21]. (**D**) Cell viability of B16F10 and human melanoma cell lines SK-Mel-37 and D10 upon stimulation with poly(I:C) and cGAMP was assessed by AlamarBlue assay. Cell lines were incubated with poly(I:C) (10 µg/mL), cGAMP (10 µg/mL) for 24 h. After 6 h of incubation with AlamarBlue (10% *v/v*), fluorescence intensity was measured. Statistical analysis was performed using an unpaired, two-tailed Students *t*-tests and the *p* values are indicated as follows: *p* > 0.05 (ns), *p* ≤ 0.05 (*), *p* ≤ 0.01 (**), *p* ≤ 0.001 (***), *p* ≤ 0.0001 (****).

To investigate if the MA gene signature is induced in activated murine macrophages, we used bone-marrow derived macrophages (BMDM) stimulated with poly(I:C) or cGAMP for 6 and 24 h in vitro. We detected a significantly enhanced expression of the signature genes *Cxcl11*, *Gbp2b* (murine homologue to *hGbp1*), and *Ido1* in stimulated BMDM at both timepoints compared to untreated cells (Figure 4A,B).

To further validate the use of the MA signature in vivo, we used the well-established murine B16F10 melanoma model. Initially, B16F10 melanoma cells were analysed for the expression of the MA signature genes after stimulation with poly(I:C) or cGAMP for 24 h in vitro. The only signature gene that was marginally upregulated in B16F10 cells upon stimulation with poly(I:C) or cGAMP was *Cxcl11* (Figure 4C). However, the magnitude of induction was approximately five-fold lower as observed in BMDM (Figure 4A,B). In addition, poly(I:C) and cGAMP were investigated for their cytotoxic effect on B16F10 melanoma cells and the human melanoma cell lines SK-Mel-37 and D10 using an AlamarBlue assay. However, cell viability was not affected in any of the tested cell lines upon incubation with poly(I:C) or cGAMP (Figure 4D).

**Figure 5:**
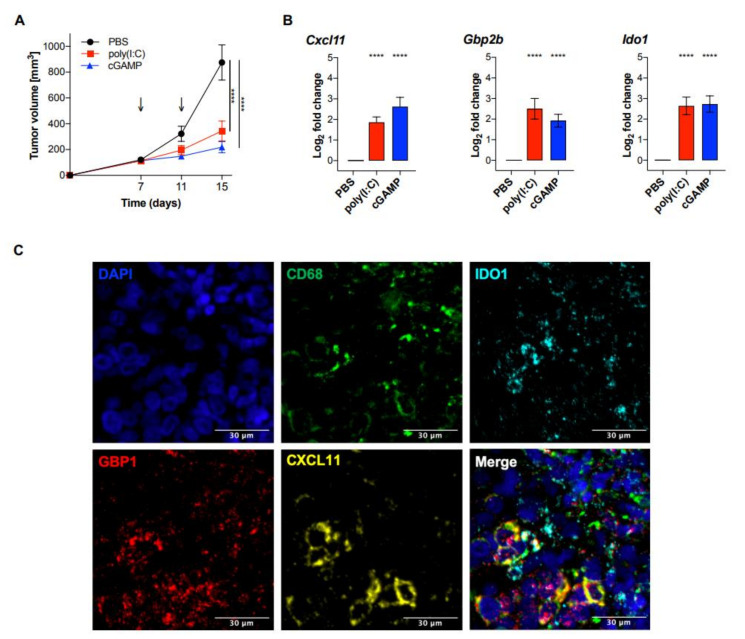
Intratumoral poly(I:C) and cGAMP significantly reduced tumor growth and induced the expression of the MA signature genes in vivo. C57BL/6J *wt* mice were injected with 2 × 10^5^ B16F10 melanoma cells and treated with poly(I:C) (50 μg/mouse), cGAMP in lipofectamine (10 μg/mouse), or PBS on day 7 and 11 after tumor injection. (**A**) Tumor size was measured using a caliper and tumor volume was calculated using the following formula: *V* = (length × width^2^)/2 (*n* = 12 per group). Statistical significance was calculated by a two-way ANOVA followed by Šidák’s multiple comparisons test. Data are represented as mean ± SEM and the *p* values are indicated as follows: *p* > 0.05 (ns), *p* ≤ 0.05 (*), *p* ≤ 0.01 (**), *p* ≤ 0.001 (***), *p* ≤ 0.0001 (****). (**B**) On day 12, tumors were isolated and analyzed for the MA signature expression by qPCR. Data were normalized to the housekeeping gene *Rplp0* and are represented as mean ± SE of log_2_ transformed values (*n* = 12 per group) [21]. Statistical analysis was performed using an unpaired, two-tailed Students *t*-tests, and the *p* values are indicated as follows: *p* > 0.05 (ns), *p* ≤ 0.05 (*), *p* ≤ 0.01 (**), *p* ≤ 0.001 (***), *p* ≤ 0.0001 (****). (**C**) Fluorescent detection of RNA transcripts in human melanoma tissue. FFPE tissue section was hybridized with Opal-labeled probes for CD68 (Opal 520), CXCL11 (Opal 570), GBP1 (Opal 620), and IDO1 (Opal 690). Nuclei were counterstained with DAPI (blue). Adjustment of brightness and color merging was performed using ImageJ. Scale bar = 30 μm.

To further explore the utility of the MA signature as a biomarker for myeloid cell activation in melanoma, B16F10 melanoma bearing C57BL/6J mice were treated with intratumoral injections of poly(I:C) or cGAMP. A significant reduction of tumor growth was observed in both treatment groups compared to control treated mice (Figure 5A).

RNA was extracted from whole tumor tissue and analyzed for the expression of *Cxcl11*, *Gbp2b*, and *Ido1*. Each of the three MA signature genes showed a significant increased expression in both poly(I:C) and cGAMP treated mice, compared to PBS treated controls (Figure 5B). Taken together, our findings suggest a potential use of the three genes *Cxcl11*, *Gbp2b*, and *Ido1* as a signature for myeloid cell activation in whole tumor tissue and will be further investigated as a clinical prognostic biomarker for melanoma patients.

## 3. Discussion

By studying the gene expression profiles of human myeloid cells upon stimulation with poly(I:C) or cGAMP, we found a gene signature associated with their activation, that comprises the three genes *Cxcl11*, *Gbp1*, and *Ido1* and allows to assess the activation state of myeloid cells in bulk tumor tissue. The myeloid activation (MA) signature positively correlated with the presence of M1 macrophages and CD8^+^ T cells within melanoma tumors, as well as improved overall survival of melanoma patients. Moreover, significantly increased expression of the MA signature was observed in on-treatment biopsies from patients with a beneficial response to PD-1 blockade.

While an in-depth study of the function of these genes and their proteins is beyond the scope of this study, we will briefly discuss the functional role of each gene. CXCL11 is a C-X-C motif chemokine that binds to the CXCR3 receptor and is involved in the recruitment, differentiation and activation of various immune cells such as monocytes, NK cells, T cells and DC [22]. In melanoma, the presence of CXCR3 ligands such as CXCL9, CXCL10, and CXCL11 has been associated with enhanced CD8^+^ T cell infiltration and hence, better survival prognosis [23].

GBP1 belongs to the GBP family of IFN-induced GTPases which play essential roles in orchestrating protective immunity to a wide range of microbes [24]. Down-regulation of GBP1 is reported to cause mitochondrial dysfunction, which eventually induces cellular senescence in inflammatory macrophages [25]. The enzyme indoleamine 2,3-dioxigenase 1 (IDO1) is a protein that catabolizes tryptophan and produces a range of kynurenine metabolites [26]. In cancers, increased IDO1 activity has been shown to promote the development of an immunosuppressive microenvironment and inhibit anti-tumor immune responses [27]. In fact, IDO1 plays a role in the suppression of effector T and NK cells and differentiation and activation of regulatory T (Treg) cells and myeloid-derived suppressor cells (MDSCs) [28,29,30]. Although IDO expression is induced by IFN-γ, TNF-α, or prostaglandins, macrophages are driven toward an immunosuppressive M2 phenotype when IDO is overexpressed [31]. In spite of the immunosuppressive action of IDO1, it has also been shown to induce inflammatory responses within the tumor microenvironment [32]. IDO1 is also involved in promoting tumor neovascularization by modulating the expression of interferon-γ (IFN-γ) and IL-6 [33,34]. However, the complete immunomodulatory effects of IDO have not yet been characterized fully. Clinical trials have targeted IDO1 with inhibitory molecules as a strategy to counteract intratumoral immunosuppression but the results from these trials have been largely inconclusive [26]. In fact, IDO1 was described as an independent prognostic marker for increased disease-free survival in patients with colorectal cancer [35]. Notably, each of the three genes constituting the MA signature has non-redundant biological functions.

In our study, we characterized a MA signature consisting of three distinct genes (*Cxcl11*, *Gbp1*, *Ido1*), which correlates with the presence of M1 macrophages and mature DC in various cancer cohorts available in TCGA. Moreover, the MA signature is positively associated with increased overall survival and beneficial treatment response in cutaneous melanoma patients.

Using the TLR3 and STING agonists poly(I:C) and cGAMP, respectively, the expression of the MA signature was induced in murine BMDM, but not in B16F10 melanoma cells. The MA signature expression was significantly elevated in murine B16F10 melanomas treated with intratumoral injection of poly(I:C) or cGAMP. Myeloid cells play a crucial role in regulating tumor immunity by altering the tumor microenvironment and regulating tumor-specific T cell recruitment [4,5,7,8]. Accordingly, increased densities of mature DC and M1 macrophages are associated with improved patient survival in a number of cancer types [36,37,38,39].

Currently, reprogramming tumor-associated myeloid cells, especially macrophages and DC, towards an immunostimulatory phenotype is a major area of investigation in the field of tumor immunology [10,40]. TLR3 targeting ligands and STING agonists are currently being tested in clinical trials as adjuvant therapy, or along with other drugs and vaccines, against a variety of cancers such as melanoma, bladder cancer, and lymphomas [11,41]. Evidence from murine models suggests that both TLR3 and STING agonists can induce macrophage polarization towards an inflammatory, anti-tumorigenic phenotype [42,43]. At the present, only a limited number of intracellular or surface markers exist for the assessment of M1 activated macrophages (e.g., iNOS, CD68, CD80) [15,44], however, as recent single-cell profiling approaches have demonstrated, canonical M1 and M2 macrophage markers are co-expressed on many subsets of tumor-associated macrophages [45]. Similarly, in-depth dissection of the tumor-infiltrating DC landscape has also revealed significant heterogeneity in phenotype and function resulting in the delineation of distinct subsets [8]. Therefore, assessing the activation state of myeloid cells remains a challenging albeit essential component of tumor immunology research both at the clinical and pre-clinical stage [46,47]. While it is possible to infer the abundance or presence of activated and resting immune cell subsets using immune deconvolution algorithms (such as CIBERSORT) in microarray or RNA-Seq datasets, these algorithms employ multigene signatures which are not feasible for the application in routine clinical tests [48]. In contrast, the MA signature described here comprises three genes that, in combination, are highly specific for activated myeloid cells and readily assessable via qPCR in tumors and by in situ hybridization of human melanoma tissues. Importantly, none of the MA signature genes were shown to be expressed in activated lymphoid cells such as T cells, B cells, or NK cells.

Recently, myeloid cell targeted therapies have entered clinical trials not only as monotherapies, but also in combination with immune checkpoint antibodies [14,40,49]. Thus, further research is required to dissect the clinical utility of myeloid cell activation therapies. While, enhanced expression of the MA signature is associated with activated M1 macrophages and improved survival in melanoma patients, it may also serve to predict the necessity and success of myeloid cell-targeted therapies under pre-clinical investigation.

The MA signature therefore offers a prognostic and diagnostic tool for both clinical researchers and basic scientists investigating the roles of myeloid cells in the immune response to tumors. In our report, we demonstrated *in situ* and *in silico*, that the MA signature is a marker for activated M1 macrophages in melanoma and other cancer types. However, whether the MA signature can also be used as a predictive marker for therapy efficacy in melanoma as well as in other malignancies is subject to further investigation.

## 4. Materials and Methods

### 4.1. Tissue Culture

Murine B16F10 melanoma cells (ATCC) were cultured in complete RPMI-1640 medium (Sigma Aldrich, Darmstadt, Germany) supplemented with 10% FBS, 100 μg/mL streptomycin, 100 U/mL penicillin, 1 mM sodium pyruvate, and 2 mM L-glutamine. Cultured B16F10 melanoma cells were plated at a concentration of 0.1–0.2 × 10^6^ cells/mL and stimulated for 24 h with poly(I:C) (Tocris Bioscience, Bristol, United Kingdom, 10 μg/mL),) cGAMP (InvivoGen, San Diego, CA, USA 10 μg/mL), or left unstimulated. BMDM were cultured using complete RPMI-1640 medium as described above. The human melanoma cell lines SK-Mel-37 and D10 were a gift from P. Zajac (University of Basel). Melanoma cells were cultured in Dulbecco’s modified Eagle’s medium (DMEM) supplemented with 10% fetal bovine serum (FBS), 100 U/mL penicillin, 1 mM sodium pyruvate, and 2 mM L-glutamine. All cells were maintained at 37 °C under 5% CO2 atmosphere.

### 4.2. Human Monocytes and DC

PBMC from healthy human donors (Interregionale Blutspende SRK) were isolated using Ficoll (GE Healthcare, Chicago, IL, USA) density gradient centrifugation. Monocytes were enriched using the EasySep^TM^ Human Monocyte Enrichment Kit w/o CD16 Depletion (STEMCELL Technologies).

DC were isolated using the MACS-based Pan-DC Enrichment Kit (Miltenyi Biotec, Bergisch Gladbach, Germany) followed by Fc-receptor blocking with anti-mouse CD16/32 (2.4G2, generated in house) for 15 min. Subsequently, cell surface markers were stained using anti-CD11c (3.9), anti-CD123 (6H6), anti-human lineage cocktail (CD3/14/16/19/20/56), anti-CD141 (M80), anti-HLA-DR (L243) (Biolegend, San Diego, CA, USA) in FACS buffer (PBS with 2% FBS and 1 mM EDTA) for 45 min on ice. Cells were then purified by FACS using the Moflo Astrios EQ cell sorter (Beckman Coulter, Nyon, Switzerland). Ethical approval provided by the Kantonale Ethikkommission Bern (KEK), Switzerland. Code: 2017-02246.

### 4.3. NanoString mRNA Profiling

Purified monocytes and DC were stimulated with poly(I:C) (Tocris Bioscience, 10 μg/mL), cGAMP (InvivoGen, 10 μg/mL) complexed with lipofectamine (3 μg/mL, Invitrogen), or left untreated for 6 h. Cells were then lysed in Buffer RLT (Qiagen, Hilden, Germany) containing 1% β-mercaptoethanol (Sigma Aldrich) and diluted 1:2 in PBS. Subsequently, 5 µL of the lysate was directly used for mRNA profiling using the NanoString Human Immunology Panel V2. Transcriptional profiling was performed using the nCounter™ Digital Analyzer (NanoString Technologies, Seattle, WA, USA). Data quality control and normalization was performed using NanoStringQCPro and NanoStringNorm. Differential gene expression was performed using edgeR package. T-distributed Stochastic Neighbor Embedding (t-SNE) was used for the dimensional reduction using the R tsne package. Permutational analysis of variance (PERMANOVA) paired test was used to test for the effect of poly(I:C) and cGAMP in both monocytes and DC cell-types.

### 4.4. BMDM Differentiation and Maturation for In Vitro Studies

To differentiate murine BMDM, bone marrow was flushed from femurs and tibias of C57BL/6J mice and cultured in petri dishes at 5 × 10^6^ cells per dish using complete RPMI-1640 (as described above) including 20% L929 supernatant and the medium was replaced on day 4. After 7 days, the BMDM were harvested and collected with a cell scraper and seeded at a concentration of 0.5 × 10^6^ cells/mL, followed by stimulation with poly(I:C) (Tocris Bioscience, 10 μg/mL), cGAMP (InvivoGen, 10 μg/mL), or left untreated for 6 and 24 h, respectively.

### 4.5. Quantitative Real-Time Polymerase Chain Reaction (qPCR)

Cells were cultured and stimulated as mentioned before and then lysed with RLT buffer (RNeasy Mini Kit, Qiagen) supplemented with 1% β-mercaptoethanol (Sigma Aldrich). Column purification was performed according to the manufacturer’s protocol (RNeasy Mini Kit, Qiagen).

For the bulk tumor analysis, total RNA was isolated in TRI Reagent (Sigma Aldrich), followed by column purification. RNA quantity was assessed using NanoDrop (ND-1000 Spectrophotometer, Thermo Scientific), followed by DNase digestion (DNase, Invitrogen) and reverse transcription of the cDNA with random primers (High Capacity cDNA Reverse Transcription Kit, Applied Biosystems) at a total RNA concentration range of 0.7–2 mg. Using the SYBR Green PCR Master Mix, 2–3 technical replicates were performed on the StepOnePlus Real-Time PCR System (ThermoFischer, Waltham, MA, USA). Applied Biosystems software provided the raw data (Ct). Relative mRNA levels were calculated by the 2^−Δ*Ct*^ method, using *Rplp0* as a housekeeping gene [21]. Primer list: Supplementary Table S3.

### 4.6. AlamarBlue Assay

Melanoma cells (SK-Mel-37, D10, B16F10) were stimulated with poly(I:C) (Tocris Bioscience, 10 μg/mL), cGAMP (InvivoGen, 10 μg/mL), or left untreated for 24 h. Following incubation of cells (B16F10, SK-Mel-37, D10) in wells (200 μL of culture medium), 10% v/v AlamarBlue (ThermoFischer) was added. After 6 h of incubation at 37 °C, fluorescence intensity was measured at 560 nm excitation and 590 nm emission using the Infinite 200 Pro Plate Reader (Tecan Life Sciences, Zürich, Switzerland). Fluorescence intensity was plotted using Graph Pad Prism 8.0.

### 4.7. Mice Tumor Inoculation and In Vivo Studies

C57BL/6 wt mice were purchased from Janvier Labs (France) and age-matched (8–10 weeks), as well as sex-matched for each experiment. Tumor inoculation was performed by subcutaneous injection of 2 × 10^5^ B16F10 melanoma cells into the left flank (day 0). After randomization, mice were treated with intratumoral (i.t.) injection of poly(I:C) (Tocris Bioscience, 50 μg/mouse) or cGAMP + LF (InvivoGen, 10 μg/mouse) and PBS as a control on day 7 and 11. Two dimensions of the tumor size were measured with a digital caliper in a blinded manner. Tumor volume was calculated as specified in the formula $V=\frac{\left(length*\;width^{2}\right)}{2}$ [50]. Mice were euthanized on day 15, followed by tumor isolation and qPCR analysis. Mice were housed in specific-pathogen free (SPF) conditions in the Central Animal Facility of the University of Bern. All animal experiments were performed according to institutional guidelines and approved by the Cantonal Veterinary Office. Ethical approval provided by the LANAT Amt für Landwirtschaft und Natur, Bern, Switzerland. Code: BE137/16.

### 4.8. RNA In Situ Hybridization (RNAscope)

The mRNA expression levels for CXCL11, GBP1, and IDO1 were investigated using the RNAscope Multiplex Fluorescent Reagent Kit v2 and the RNAscope 4-Plex Ancillary Kit for Multiplex Fluorescent Kit v2, Advanced Cell Diagnostics, Inc. (ACD, Hayward, CA). The following probes were used: Hs-CD68-C4, Hs-CXCL11-C3, Hs-GBP1, Hs-IDO1-C2. For fluorescent detection, the label probes were conjugated to Opal 520 (CD68, 1:800), Opal 570 (CXCL11, 1:1500), Opal 620 (GBP1, 1:1500), and Opal 690 (IDO1, 1:800) from Akoya Biosciences/PerkinElmer (Waltham, MA, USA). The assay was performed according to the manufacturer’s instructions. Briefly, FFPE tissue slides were deparaffinizing and pre-treated before progressing onto hybridization with target probes. For the hybridization, slides were covered in a HybEZ™ Humidity Control Tray and placed in the HybEZ™ Oven and incubated at 40 °C for 2 h. Hybridization with target probes, preamplifier, amplifier, and label probes were performed according to manufacturer’s instructions. Nuclear staining was performed using DAPI. Images were acquired using the Akoya Vectra 3.0 Automated Quantitative Pathology Imaging System (P/N CLS142568) and analyzed using with Phenochart™ and inForm (Akoya Biosciences, Menlo Park, CA, USA). Adjustment of brightness and color merging of TIFF format files was performed using ImageJ.

### 4.9. TCGA Data Analysis

TCGA RNA-seq gene expression data as well as clinical information from all available cancer cohorts were obtained using the package GDCRNATools in R [51]. Raw counts were transformed into transcripts per million (TPM), which normalizes by gene lengths. Gene expression of *Cxcl11*, *Gbp1*, and *Ido1* was used to assign a score to every patient based on a non-parametric, rank-based method implemented in the singscore R package. The score allowed for patients’ stratification into two groups according to their MA signature expression (low or high split by the sample median).

Kaplan–Meier curves were plotted using the R survminer package. The survival curves were compared using the log-rank test. The M1 signature and mature DC signature scores were calculated using the singscore R package [17,18]. Correlation between the MA signature score and the M1 signature and mature DC based scores were tested using Pearson correlation. The relative proportions of 22 types of infiltrating immune cell subsets in melanoma with high vs. low MA signature expression were determined via the LM22 leukocyte signature matrix using CIBERSORT [52].

### 4.10. Analysis of GEO Datasets

Publicly available datasets (GSE2706, GSE57494, GSE1925, GSE82227, GSE60235, GSE79828, GSE85543, GSE63038, GSE107011) were obtained from gene expression omnibus (GEO) using GEOquery package. Microarrays were individually background corrected and normalized using R limma package. RNA-seq data used log2 FPKM values. Genes with multiple probes or transcripts were aggregated to the sample median. Ultimately, linear models were used to assess the association between gene expression and classical innate immune stimuli such as LPS and IFNγ, adjusting for “time” and “sample pairing” if relevant.

### 4.11. Statistical Analysis

Statistical analyses were performed using GraphPad Prism 8.0 (GraphPad Software) or R (R-project CRAN). Statistical significance was represented as described in the figure legends.

## 5. Conclusions

In this study, we characterized a myeloid activation (MA) signature comprising the three genes *Cxcl11*, *Gbp1*, and *Ido1*. The MA signature indicates the activation state of myeloid cells and represents a prognostic biomarker for the overall survival in melanoma patients.

## Figures and Tables

**Table 1:** Signed fold-change table of common genes that were differentially expressed upon cGAMP or poly(I:C) stimulation in both monocytes and dendritic cells.

Genes	Monocytes		DC	
	cGAMP	Poly(I:C)	cGAMP	Poly(I:C)
*CCL3*	5.26	5.48	3.7	4.83
*CCL4*	4.97	4.87	3.62	5.14
*CXCL10*	9.10	3.89	10.69	7.40
*CXCL11*	9.96	3.42	11.21	8.96
*GBP1*	4.79	2.31	4.37	3.91
*IDO1*	6.82	4.26	2.67	3.41
*IFNB1*	17.73	6.95	16.71	15.18
*IL27*	15.05	10.80	11.03	11.54
*IL-29*	13.44	8.86	15.03	14.28
*TGFBR2*	−3.73	−3.89	−9.72	−9.91

Signed fold-change table of differentially expressed genes (DEG) that were common between human DC (*n* = 4–5) and CD14+ monocytes (*n* = 4–5) upon stimulation with cGAMP or poly(I:C). Gene expression was quantified using the NanoString nCounter Immunology.

**Table 2:** *p*-value table from differential gene expression tests between unstimulated versus stimulated myeloid cells in RNA-seq and microarray datasets.

Genes	RNA-seq		Microarray	
	GSE57494	GSE82227	GSE2706	GSE1925
*CCL3*	<0.001	1.000	0.003	0.137
*CCL4*	<0.001	1.000	0.001	0.027
*CXCL10*	<0.001	<0.001	<0.001	0.074
*CXCL11*	<0.001	<0.001	0.010	<0.001
*GBP1*	<0.001	<0.001	0.001	<0.001
*IDO1*	<0.001	<0.001	<0.001	0.001
*IFNB1*	0.003	1.000	1.000	1.000
*IL27*	<0.001	<0.001	0.013	NA
*IL-29*	0.041	NA	1.00	NA
*TGFBR2*	<0.001	0.014	0.009	0.433

*p*-value table from differentially expressed genes between stimulated and unstimulated myeloid cells derived from GEO datasets comprising RNA-seq or microarray gene expression data. Data sets include circulating myeloid DC (GSE2706), primary human CD14^+^/CD16^+^ monocytes (GSE57494), CD14^+^ monocytes (GSE1925), and CD14^+^ cells (GSE82227). Data were normalized and *p*-value was computed by regression analysis. Not available (NA).

## Supplementary Materials

The following are available online at https://www.mdpi.com/2072-6694/12/6/1431/s1, Figure S1: Correlation between CXCL11, GBP1, and IDO1 signature score and abundance of immune cells deconvoluted with CIBERSOR., Figure S2: Signature Score in melanoma patients before anti-PD-1 treatment, Table S1: Expression of MA signature genes Cxcl11, Gbp1 and Ido1 in B cells, NK cell, CD4 and CD8 T cells upon activation., Table S2: TCGA study abbreviations and corresponding study names, Table S3: Primer list.
