# Supplementary material for: Characterization of a Myeloid Activation Signature That Correlates with Survival in Melanoma Patients
Source: Cancers (Basel). 2020 May 31;12(6):1431. doi: 10.3390/cancers12061431 (PMC7352688; doi:10.3390/cancers12061431)
Supplement: Supplementary file 1 [file cancers-12-01431-s001.pdf]

Supplementary Materials

Characterization of a Myeloid Activation Signature that Correlates with Survival in Melanoma Patients

Mirela Kremenovic, Nives Rombini, Alfred A. Chan, Thomas Gruber, Lukas Bäriswyl, Delphine J. Lee and Mirjam Schenk

Table S1. Expression of MA signature genes *Cxcl11*, *Gbp1* and *Ido1* in B cells, NK cell, CD4 and CD8 T cells upon activation.

Heading Title	CD4 T Cells (GSE60235)	CD8 T Cells (GSE79828)	B Cells (GSE85543)	NK Cells (GSE63038)
**Activation stimuli**:	aCD3/aCD28	aCD3/aCD28	sCD40L	IL12, IL12/IgG
CXCL11	No	No	No	No
GBP1	No	No	No	No
IDO1	No	No	No	No

GEO datasets (GSE60235, GSE79828, GSE85543, GSE63038) containing microarray data of $CD4^+$ T cells (n = 15), $CD8^+$ T cells (n = 4), naïve B cells (n = 6), and $CD56^{dim}CD16^+$ NK cells (n = 8) after 6 to 48h of cell specific activation were normalized with RMA using the *oligo* (Affymetrix arrays) or the *agilp* packages in R (Agilent arrays) and analysed. Paired *t*-test was performed for every signature gene between stimulated and unstimulated samples. The attribute "*Yes*" was assigned if the analysed gene was upregulated with the following thresholds in a given dataset; $\log_2 FC \geq 2$, p value < 0.05, otherwise the attribute "*No*" was assigned.

Table S2. TCGA study abbreviations and corresponding study names.

Study Abbreviation	Study Name
LAML	Acute myeloid leukemia
ACC	Adrenocortical carcinoma
BLCA	Bladder urothelial carcinoma
LGG	Brain lower grade glioma
BRCA	Breast invasive carcinoma
CESC	Cervical squamous cell carcinoma and endocervical adenocarcinoma
CHOL	Cholangiocarcinoma
COAD	Colon adenocarcinoma
ESCA	Esophageal carcinoma
GBM	Glioblastoma multiforme
HNSC	Head and neck squamous cell carcinoma
KICH	Kidney chromophobe
KIRC	Kidney renal clear cell carcinoma
KIRP	Kidney renal papillary cell carcinoma
LIHC	Liver hepatocellular carcinoma
LUAD	Lung adenocarcinoma
LUSC	Lung squamous cell carcinoma
DLBC	Lymphoid neoplasm diffuse large B-cell lymphoma
MESO	Mesothelioma
OV	Ovarian serous cystadenocarcinoma
PAAD	Pancreatic adenocarcinoma
PCPG	Pheochromocytoma and paraganglioma
PRAD	Prostate adenocarcinoma
READ	Rectum adenocarcinoma
SARC	Sarcoma
SKCM	Skin cutaneous melanoma
STAD	Stomach adenocarcinoma
TGCT	Testicular germ cell tumors
THYM	Thymoma
THCA	Thyroid carcinoma
UCS	Uterine carcinosarcoma
UCEC	Uterine corpus endometrial carcinoma
UVM	Uveal melanoma

Table S3. Primer list.

Gene	Primer Sequence
Cxcl11	Fwd: 5′-TGC GAC AAA GTT GAA GTG ATT GTT-3′<br>Rev: 5′-ATA CGT GGC TGC ATG TTC CA-3′
Gbp1	Fwd: 5′-AAC TTC AGG AAC AGG AAA GAC TTC-3′<br>Rev: 5′-ACA ATC CAA AGC TGT CCC CG-3′
Ido1	Fwd: 5′-AGG ATG CGT GAC TTT GTG GA-3′<br>Rev: 5′-TCC CAG ACC CCC TCA TAC AG-3′
Rplp0	Fwd: 5′-GGA CCG CCT GGT TCT CCT AT-3′<br>Rev: 5′-ACG ATG TCA CTC CAA CGA GG-3′

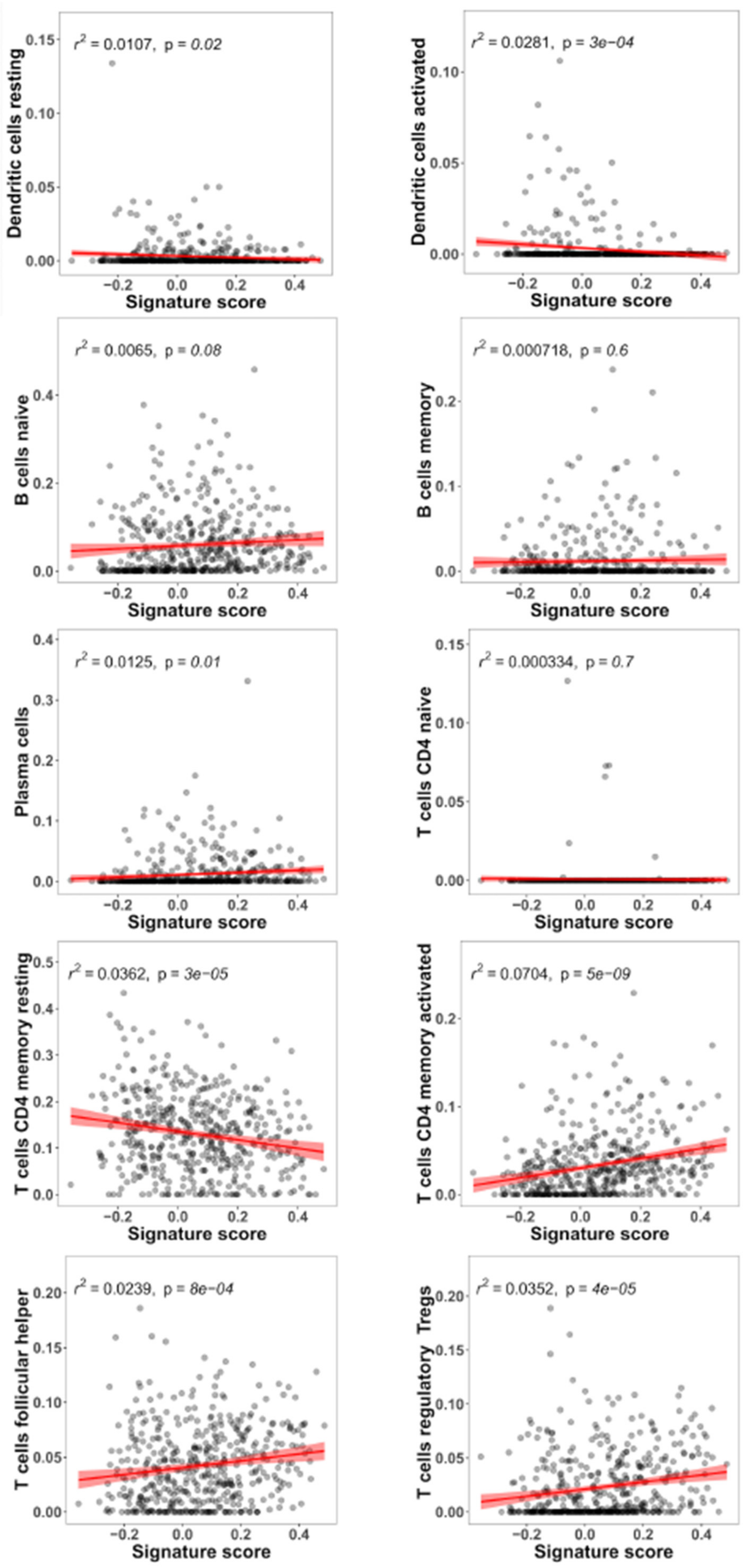
r² = 0.0107, p = 0.02
Dendritic cells resting
Signature score
r² = 0.0281, p = 3e−04
Dendritic cells activated
Signature score
r² = 0.0065, p = 0.08
B cells naive
Signature score
r² = 0.000718, p = 0.6
B cells memory
Signature score
r² = 0.0125, p = 0.01
Plasma cells
Signature score
r² = 0.000334, p = 0.7
T cells CD4 naive
Signature score
r² = 0.0362, p = 3e−05
T cells CD4 memory resting
Signature score
r² = 0.0704, p = 5e−09
T cells CD4 memory activated
Signature score
r² = 0.0239, p = 8e−04
T cells follicular helper
Signature score
r² = 0.0352, p = 4e−05
T cells regulatory Tregs
Signature score

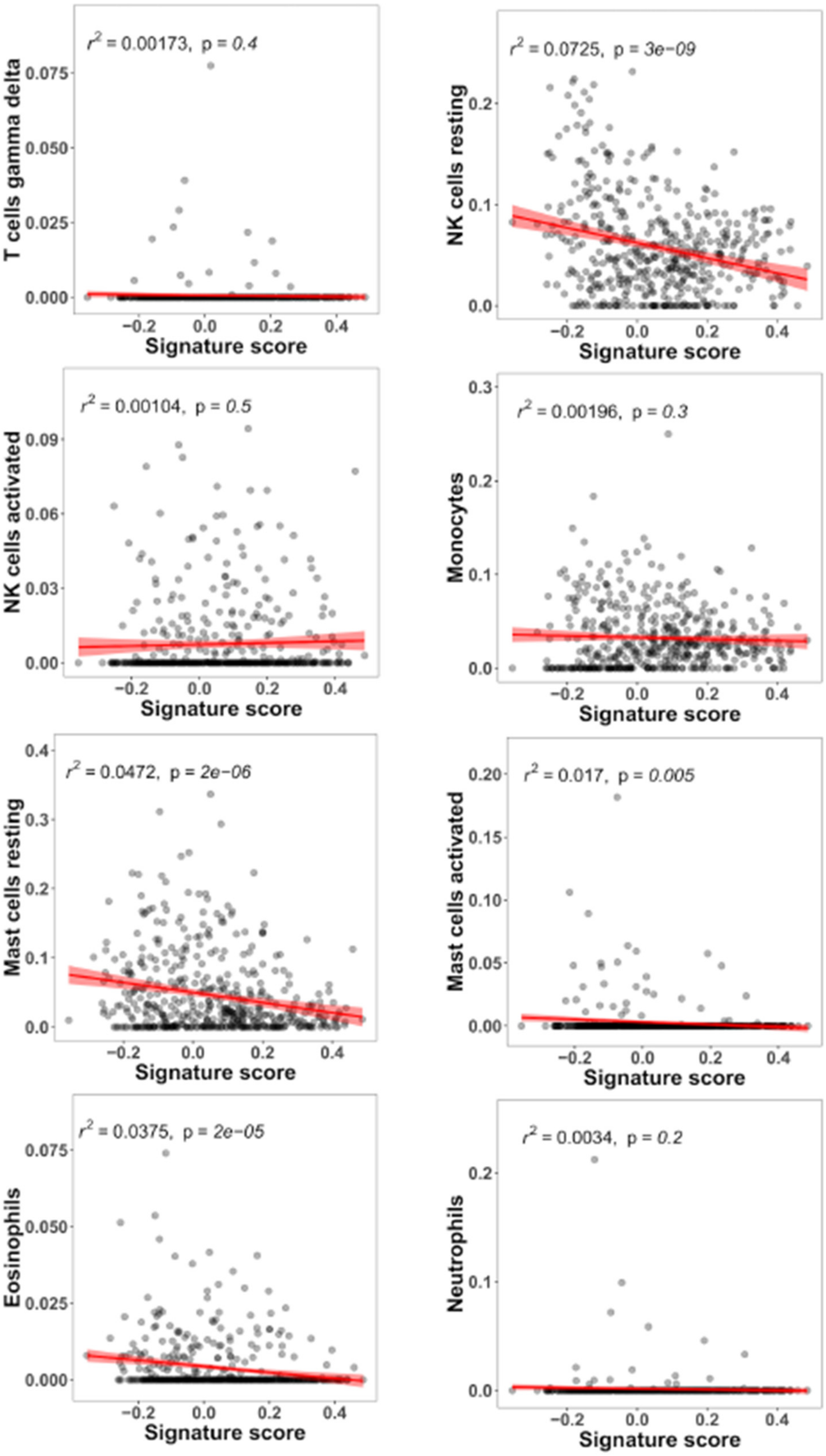

Figure S1. Correlation between *CXCL11*, *GBP1*, and *IDO1* signature score and abundance of immune cells deconvoluted with CIBERSORT**.** Pearson correlation of *CXCL11*, *GBP1*, and *IDO1* score assigned to SKCM-TCGA patients and the estimated abundances of 16 out of 22 immune cells determined by CIBERSORT. Each dot represents one patient.

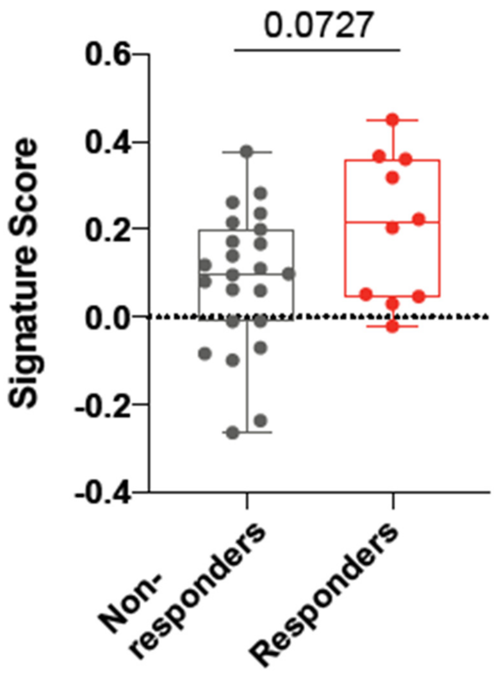

Figure S2. Signature Score in melanoma patients before anti-PD- 1 treatment. Patients were stratified into responders (complete response and partial response, n = 10) and non-responders (progressive disease, n = 23). Each dot represents an individual patient and only patients sampled pre- and post-treatment were included in the analysis. The dataset was obtained from GSE91061. Box plot defines the maximum, third quartile, first quartile and minimum values. p-values were determined by two-sided Welch's t-test (* $p < 0.0332$; ** $p < 0.0021$; *** $p < 0.0002$; **** $p < 0.0001$).

© 2020 by the authors. Licensee MDPI, Basel, Switzerland. This article is an open access article distributed under the terms and conditions of the Creative Commons Attribution (CC BY) license (http://creativecommons.org/licenses/by/4.0/).
